# Supplementary material for: A Computational Study on the Mechanism of Catalytic Cyclopropanation Reaction with Cobalt N-Confused Porphyrin: The Effects of Inner Carbon and Intramolecular Axial Ligand
Source: Molecules. 2022 Oct 26;27(21):7266. doi: 10.3390/molecules27217266 (PMC9658855; doi:10.3390/molecules27217266)
Supplement: Supplementary file 1 [file molecules-27-07266-s001.zip › molecules-1912336-supplementary.pdf]

Table of contents

Table of contents……………………………………………………………………...………………….S1

1. Optimized structures of Co(NCTPPSpy)……………………………...………………...…..……..S2

2. Calculated energy diagrams……………………………………………………….……..S3–S5

3. Summary table of energy diagram………………...…………………………………………S6

4. Equation of reaction rate in TS1……………..……………………………………………….S6

5. Calculated Mulliken spin populations..……..…………………………...……………S7–S8

6. Cartesian coordinates…………………..……..…………………………………………S9–S41

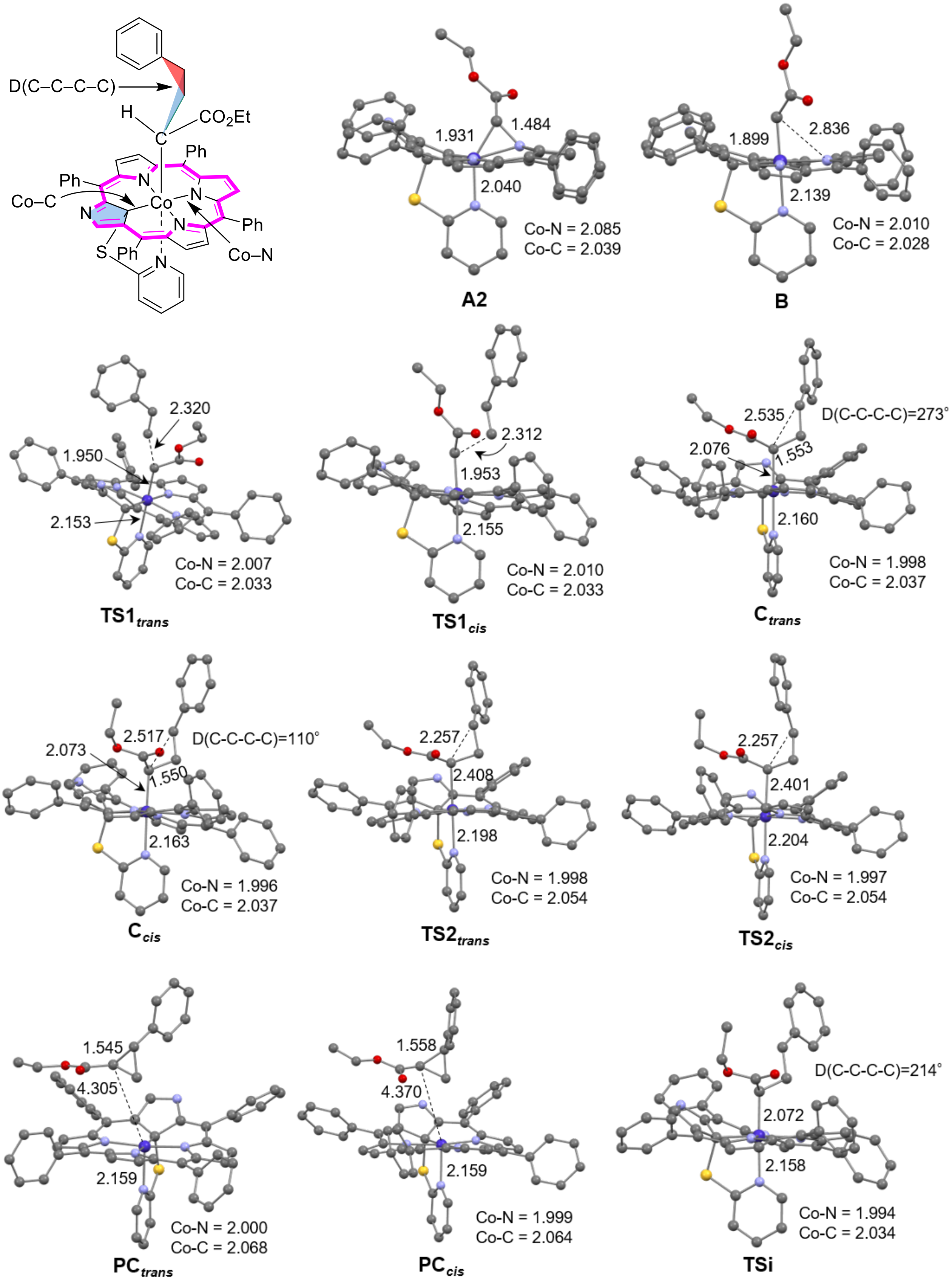

Figure S1. Optimized structures of reaction species and transition states in **Co(NCTPPSpy)**.

The units are in Å. Hydrogen atoms are omitted for clarity.

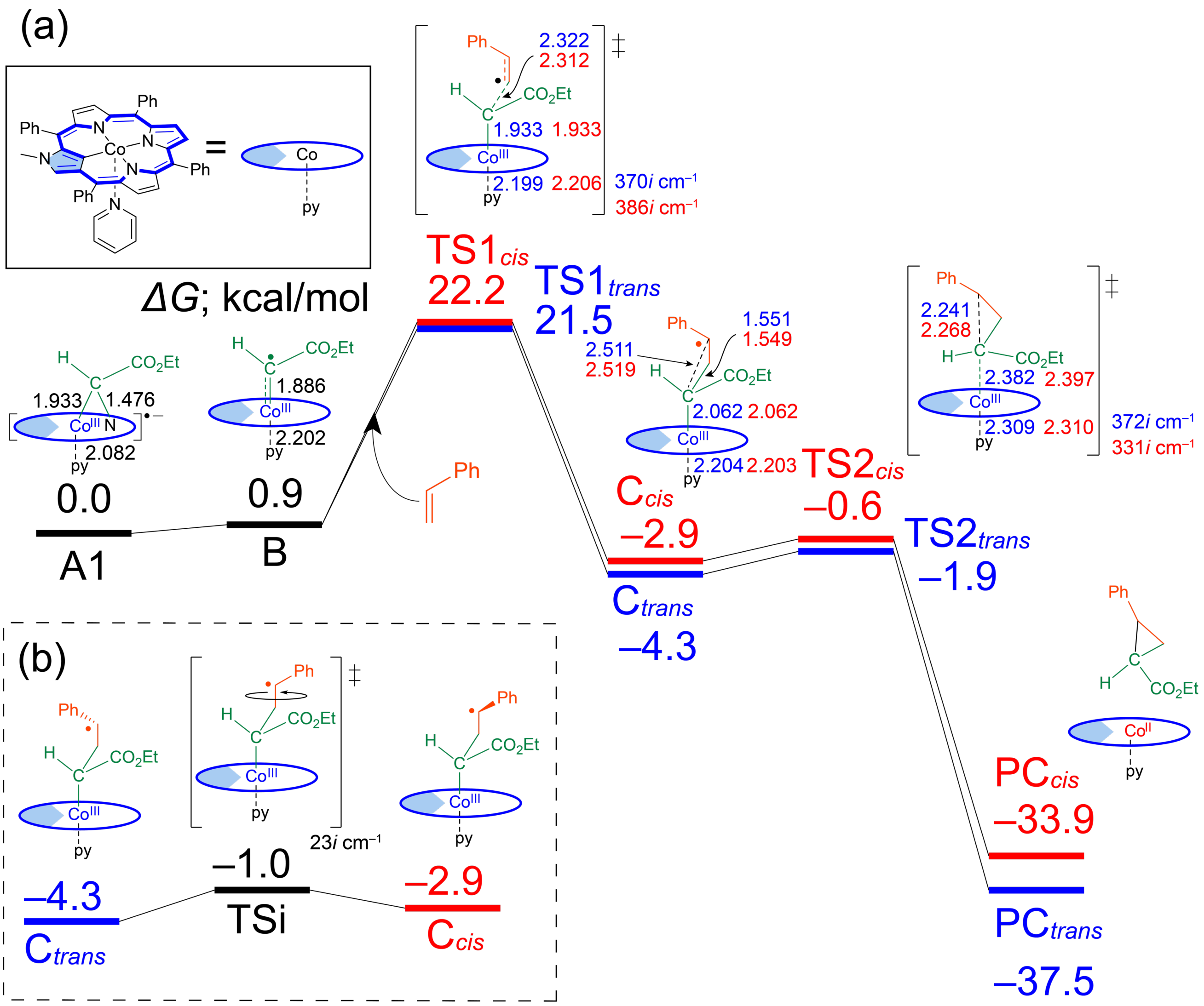

Figure S2. (a) Energy profile for cyclopropanation reaction catalyzed by **Co(MeNCTPP)(py)**. (b) Interconversion between **C*trans*** and **C*cis***. The relative Gibbs free energies (kcal/mol) are shown with respect to **A1**. Bond lengths (Å), Blue: *trans* route, Red: *cis* route.

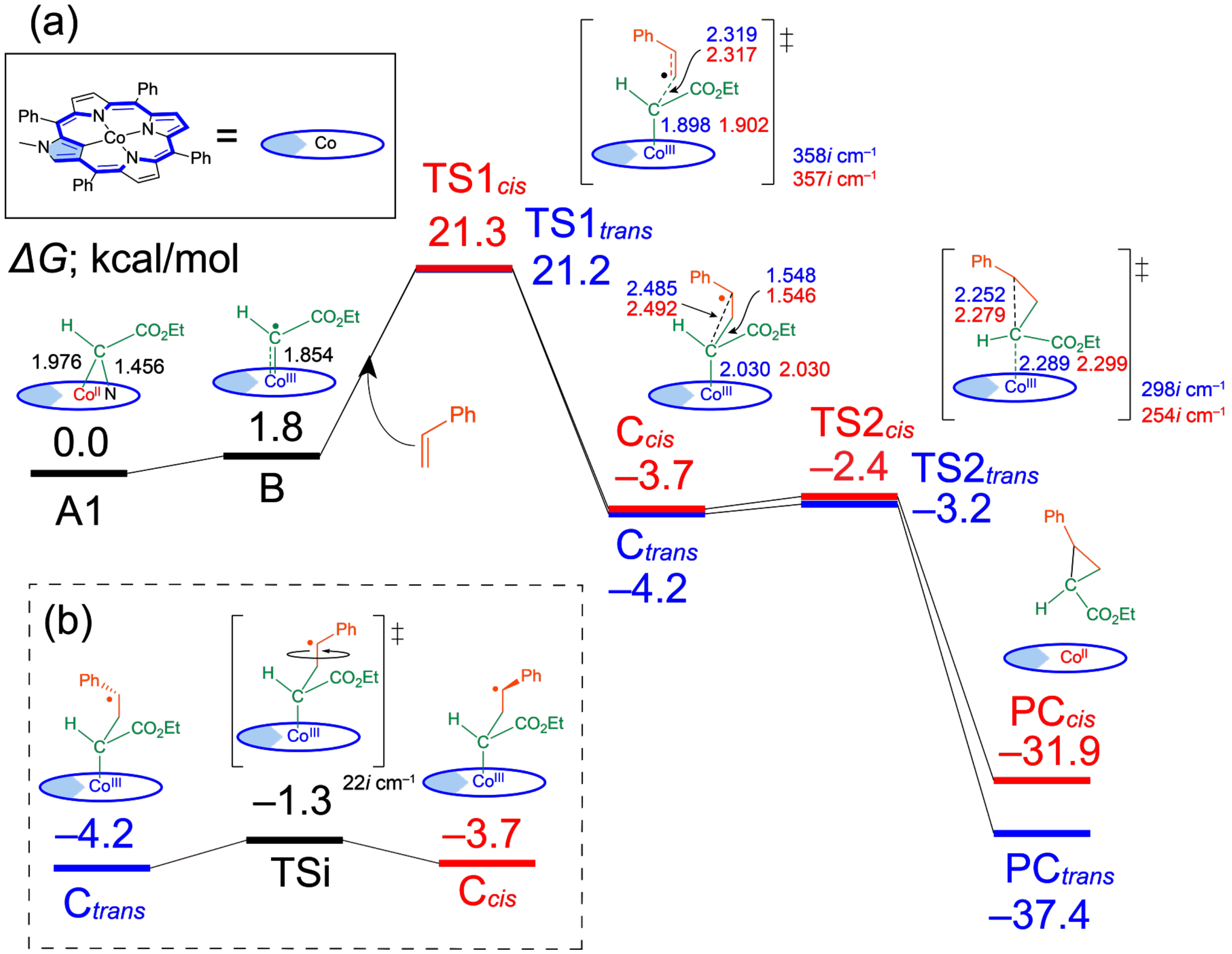

Figure S3. (a) Energy profile for cyclopropanation reaction catalyzed by **Co(MeNCTPP)**. (b) Interconversion between $\mathbf{C}_{\boldsymbol{trans}}$ and $\mathbf{C}_{\boldsymbol{cis}}$. The relative Gibbs free energies (kcal/mol) are shown with respect to **A1**. Bond lengths (Å), Blue: *trans* route, Red: *cis* route.

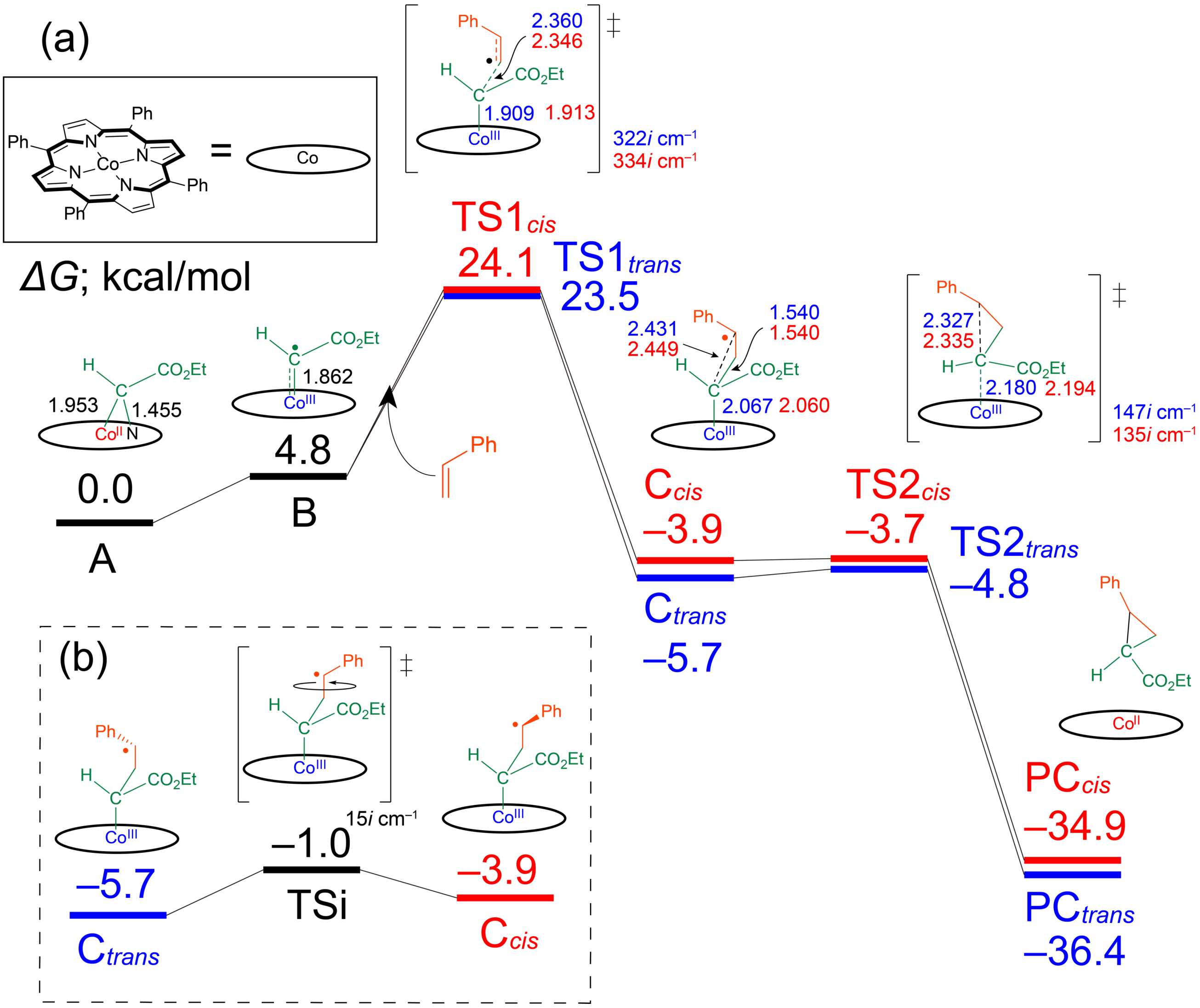

Figure S4. (a) Energy profile for cyclopropanation reaction catalyzed by **Co(TPP)**. (b) Interconversion between **C*trans*** and **C*cis***. The relative Gibbs free energies (kcal/mol) are shown with respect to **A**. Bond lengths (Å), Blue: *trans* route, Red: *cis* route.

Table S1. Relative Gibbs free energies *ΔG* of *trans*-species with respect to **A1** or **A2** are in kcal/mol. Those of *cis* species are shown in the parentheses.

Catalyst	A	B	TS1$_{trans}$ (TS1$_{cis}$)	C$_{trans}$ (C$_{cis}$)	TS2$_{trans}$ (TS2$_{cis}$)	PC$_{trans}$ (PC$_{cis}$)	TSi
**Co(TPP)**	—	4.8	23.5 (24.1)	–5.7 (–3.9)	–4.8 (–3.7)	–36.4 (–34.9)	–1.0
**Co(MeNCTPP)**	**A1**<br>[**A2**: +5.3]	1.8	21.2 (21.3)	–4.2 (–3.7)	–3.2 (–2.4)	–37.4 (–31.9)	–1.3
**Co(MeNCTPP)(py)**	**A1**<br>[**A2**: +7.4]	0.9	21.5 (22.2)	–4.3 (–2.9)	–1.9 (–0.6)	–37.5 (–33.9)	–1.0
**Co(NCTPPSpy)**	**A2**<br>[**A1**: +1.1]	0.4	22.7 (23.3)	–2.0 (–1.6)	0.4 (0.4)	–35.4 (–31.5)	–0.1

The equation of reaction rate in TS1

The reaction rate in **TS1** is calculated by Arrhenius equation and the relational expression between equilibrium constant and free energy as shown in below equations (1)–(4).

$$v = k[\text{styrene}][\mathbf{B}] \qquad (1)$$

$$k = A \exp\left[-\frac{E_a(\text{TS1})}{RT}\right] \qquad (2)$$

$$K = \frac{[\mathbf{B}]}{[\mathbf{A}]} = \exp\left[-\frac{\Delta G(\mathbf{B}-\mathbf{A})}{RT}\right] \qquad (3)$$

$$v = A[\text{styrene}][\mathbf{A}] \exp\left[-\frac{\Delta G(\mathbf{B}-\mathbf{A}) + E_a(\text{TS1})}{RT}\right] \qquad (4)$$

where *v* is the reaction rate, *k* is the rate constant, *A* is the pre-exponential factor, *R* is the universal gas constant, *T* is the absolute temperature, *K* is the equilibrium constant between **A** and **B**, *ΔG*(**B**–**A**) is the relative Gibbs free energy, and E_a is the activation free energy for **TS1** relative to **B**. The concentration of styrene and **A** ([styrene] and [**A**], respectively) can be regarded as the constant value. Therefore, the reaction rate is depended on *ΔG*(**B**–**A**) and E_a(TS1), according to equation (4).

Table S2. Calculated Mulliken spin populations in **Co(NCTPPSpy)**.

Species	Co	C (carbene)	C (benzyl)
**A2**	0.30	–0.03	—
**B**	–0.06	0.91	—
**TS1*trans***	–0.02	0.68	0.37
**TS1*cis***	–0.02	0.66	0.38
**C*trans***	0.08	–0.01	0.73
**C*cis***	0.09	–0.02	0.72
**TS2*trans***	0.73	–0.36	0.55
**TS2*cis***	0.74	–0.36	0.53
**PC*trans***	1.01	0.00	0.00
**PC*cis***	1.01	0.00	0.00

Table S3. Calculated Mulliken spin populations in **Co(MeNCTPP)(py)**.

Species	Co	C (carbene)	C (benzyl)
**A1**	0.10	–0.05	—
**B**	–0.03	0.88	—
**TS1*trans***	0.02	0.64	0.36
**TS1*cis***	0.04	0.60	0.37
**C*trans***	0.09	–0.01	0.72
**C*cis***	0.08	–0.01	0.72
**TS2*trans***	0.77	–0.36	0.55
**TS2*cis***	0.78	–0.38	0.55
**PC*trans***	1.06	0.00	0.00
**PC*cis***	1.06	0.00	0.00

Table S4. Calculated Mulliken spin populations in **Co(MeNCTPP)**.

**Species**	**Co**	**C (carbene)**	**C (benzyl)**
**A1**	1.08	–0.03	—
**B**	0.01	0.85	—
**TS1*trans***	0.19	0.53	0.35
**TS1*cis***	0.17	0.54	0.35
**C*trans***	0.22	–0.08	0.71
**C*cis***	0.21	–0.08	0.71
**TS2*trans***	0.82	–0.34	0.55
**TS2*cis***	0.83	–0.35	0.54
**PC*trans***	1.14	0.00	0.00
**PC*cis***	1.14	0.00	0.00

Table S5. Calculated Mulliken spin populations in **Co(TPP)**.

**Species**	**Co**	**C (carbene)**	**C (benzyl)**
**A**	1.03	–0.04	—
**B**	–0.06	0.92	—
**TS1*trans***	0.03	0.68	0.34
**TS1*cis***	0.04	0.66	0.35
**C*trans***	0.41	–0.19	0.67
**C*cis***	0.39	–0.18	0.67
**TS2*trans***	0.67	–0.31	0.60
**TS2*cis***	0.69	–0.32	0.58
**PC*trans***	1.09	0.00	0.00
**PC*cis***	1.10	0.00	0.00

Table S6. Cartesian coordinates of **A2** in the doublet state of **Co(NCTPPSpy)**.

	Angstrom		
Atom	X	Y	Z
N	-0.180170	-1.845885	0.390015
C	0.835303	-2.769382	0.492583
C	-1.351398	-2.553649	0.570072
C	0.310655	-4.053473	0.874778
H	0.896481	-4.948861	1.029862
C	-1.054282	-3.919376	0.916972
H	-1.791816	-4.675575	1.148131
N	1.909042	-0.161250	-0.708064
C	2.651991	-1.386383	-0.488555
C	2.908670	0.883919	-0.794385
C	4.192008	0.255044	-0.889383
H	5.125685	0.794142	-0.973963
C	4.035643	-1.093381	-0.694363
H	4.824008	-1.827359	-0.596978
N	0.273626	2.131852	0.058607
C	1.449447	2.808359	-0.156970
C	-0.693227	3.108651	0.245731
C	1.251187	4.225005	-0.005762
H	2.014259	4.981680	-0.122790
C	-0.084794	4.409117	0.257624
H	-0.606332	5.341852	0.422669
C	-1.909734	0.356710	0.633096
C	-2.852546	-0.696484	0.134227
C	-2.593330	1.584569	0.143064
C	-3.807230	1.156831	-0.479576
H	-4.529177	1.805008	-0.969131
N	-3.954113	-0.161349	-0.503906
C	2.172867	-2.557982	0.076725
C	-2.657278	-2.064279	0.267029
C	-2.094364	2.879950	0.166497
C	2.687529	2.232765	-0.579396
Co	0.080284	0.131662	0.249641
C	4.915435	-5.881929	0.348847
C	4.480831	-5.419109	-0.902403
C	3.594169	-4.336987	-0.985993
C	3.127406	-3.706541	0.182347
C	3.559884	-4.180716	1.433661
C	4.452097	-5.258661	1.517162
H	4.828222	-5.903076	-1.813267
H	3.241010	-3.983030	-1.952254
H	3.200938	-3.698565	2.341011

H	4.783648	-5.611178	2.492045
C	-5.901312	-4.806671	-0.502102
C	-6.091313	-3.696644	0.334911
C	-5.038319	-2.808682	0.579136
C	-3.767660	-3.017417	0.005446
C	-3.589974	-4.135475	-0.836059
C	-4.646154	-5.018572	-1.090806
H	-7.061880	-3.521301	0.795210
H	-5.192736	-1.948674	1.224968
H	-2.624471	-4.300324	-1.308950
H	-4.489601	-5.869115	-1.751703
C	-4.883635	6.136810	-0.343724
C	-5.068680	5.188928	0.673018
C	-4.156165	4.137991	0.829715
C	-3.033852	4.022780	-0.015859
C	-2.859295	4.981127	-1.035273
C	-3.777851	6.024683	-1.200446
H	-5.922384	5.267027	1.343406
H	-4.297838	3.407989	1.623719
H	-2.015119	4.889930	-1.714475
H	-3.633962	6.746838	-2.001835
C	6.097230	4.843769	-1.094932
C	5.536314	4.657716	0.177630
C	4.426690	3.818471	0.343371
C	3.862727	3.146056	-0.757306
C	4.434998	3.335872	-2.028530
C	5.540766	4.180595	-2.198218
H	5.962831	5.164551	1.041189
H	3.997650	3.674919	1.333007
H	4.003390	2.826387	-2.887924
H	5.965043	4.320823	-3.190548
S	-2.098418	0.360580	2.507640
C	2.137919	0.322123	4.043354
C	1.082271	0.410768	4.969932
C	-0.228755	0.426856	4.506495
C	-0.458129	0.348573	3.113985
N	0.551214	0.258844	2.230138
C	1.818777	0.249913	2.690979
H	3.177258	0.309712	4.357654
H	1.284305	0.469524	6.037550
H	-1.070686	0.499986	5.189204
H	2.599934	0.182635	1.939739
C	0.705802	-0.047318	-1.568665
C	0.353599	-1.152815	-2.496124

C	-1.287411	-1.772980	-4.103882
C	-2.573853	-1.205098	-4.685023
O	0.920312	-2.224389	-2.651251
H	0.685965	0.913600	-2.076420
H	-1.468880	-2.703919	-3.554832
H	-0.542604	-1.981809	-4.881790
H	-3.018206	-1.929231	-5.378145
H	-3.296306	-0.996365	-3.889166
H	-2.379568	-0.276105	-5.232312
H	5.604983	-6.721508	0.413120
H	6.957091	5.498010	-1.224754
H	-5.594432	6.951166	-0.470422
H	-6.721931	-5.494944	-0.696092
O	-0.752187	-0.787881	-3.191588

Table S7. Cartesian coordinates of **B** in the doublet state of **Co(NCTPPSpy)**.

Atom	X	Y	Z
	Angstrom		
N	-1.939719	0.795534	-0.162515
C	-2.273292	2.134398	-0.096003
C	-3.132290	0.114469	-0.240493
C	-3.707018	2.299939	-0.238369
H	-4.226093	3.248542	-0.242732
C	-4.237526	1.048846	-0.338845
H	-5.274975	0.769112	-0.457913
N	0.689899	1.896169	0.069248
C	-0.008705	3.045045	0.330894
C	2.017951	2.232339	0.132107
C	2.166033	3.623642	0.502120
H	3.108789	4.134421	0.643290
C	0.902785	4.132560	0.621840
H	0.610606	5.143969	0.869179
N	1.764635	-0.701910	-0.429194
C	2.928933	0.028172	-0.524363
C	2.136071	-2.014998	-0.584850
C	4.037995	-0.836779	-0.869237
H	5.051906	-0.506059	-1.048281
C	3.544941	-2.108633	-0.902047
H	4.071587	-3.027165	-1.122381
C	-0.843172	-1.773589	-0.624549
C	-2.193384	-2.120095	-0.061129
C	-0.058862	-2.966364	-0.173281
C	-0.977982	-3.843514	0.460981
H	-0.736577	-4.795957	0.923393
N	-2.219266	-3.355042	0.523901
C	-1.403797	3.193486	0.192841
C	-3.308177	-1.283679	-0.067414
C	1.310154	-3.140050	-0.320088
C	3.084580	1.385682	-0.211214
Co	-0.072379	0.062900	-0.243366
C	-3.066295	7.144959	0.754949
C	-2.644876	6.389933	1.859526
C	-2.106194	5.109192	1.674391
C	-1.984835	4.562653	0.383987
C	-2.411255	5.325273	-0.718084
C	-2.946962	6.607969	-0.535235
H	-2.736485	6.795730	2.865168
H	-1.785339	4.523418	2.533617

H	-2.316085	4.913575	-1.721097
H	-3.268892	7.186291	-1.399177
C	-7.207666	-2.952503	0.729973
C	-6.393002	-3.491630	-0.277194
C	-5.127785	-2.948603	-0.525688
C	-4.657468	-1.845735	0.214291
C	-5.481627	-1.316038	1.227313
C	-6.743377	-1.866078	1.485536
H	-6.742211	-4.337782	-0.865891
H	-4.497959	-3.372486	-1.303872
H	-5.119525	-0.488615	1.833357
H	-7.359669	-1.450597	2.280540
C	3.028082	-7.056042	0.265305
C	1.972057	-6.871832	-0.639154
C	1.419192	-5.598772	-0.826015
C	1.919181	-4.484118	-0.122010
C	2.981169	-4.681550	0.784543
C	3.527359	-5.955772	0.978826
H	1.580094	-7.717861	-1.200296
H	0.608778	-5.457907	-1.537899
H	3.359771	-3.836162	1.354448
H	4.338633	-6.090846	1.691472
C	7.089508	2.984222	0.023735
C	6.254377	3.348073	-1.042686
C	4.952080	2.834440	-1.123969
C	4.473417	1.942625	-0.148228
C	5.316680	1.580764	0.919219
C	6.614708	2.101666	1.005774
H	6.614759	4.030152	-1.810404
H	4.307080	3.117081	-1.953842
H	4.939643	0.903733	1.682774
H	7.254054	1.819397	1.840105
S	-1.035227	-1.997828	-2.479589
C	0.344282	1.891191	-4.268289
C	-0.115568	0.872104	-5.120538
C	-0.542541	-0.327552	-4.562814
C	-0.496373	-0.478500	-3.158134
N	-0.055943	0.492372	-2.339148
C	0.353031	1.650552	-2.897202
H	0.687544	2.847532	-4.651805
H	-0.140621	1.014502	-6.199034
H	-0.908913	-1.142329	-5.181131
H	0.697371	2.409220	-2.204487
C	-0.168547	-0.321915	1.614072

C	0.875049	-0.245412	2.621511
H	-1.143166	-0.600943	2.018915
H	-3.482884	8.140056	0.898112
H	8.099297	3.384899	0.089297
H	3.455560	-8.045679	0.414499
H	-8.189501	-3.378461	0.928046
O	0.374815	-0.565313	3.868629
C	1.319353	-0.517681	4.951170
C	0.574530	-0.878709	6.229959
O	2.061566	0.057502	2.472663
H	1.757423	0.486951	5.009873
H	2.138945	-1.220394	4.752226
H	1.262466	-0.856618	7.083869
H	-0.237595	-0.168713	6.420879
H	0.142250	-1.882732	6.158666

Table S8. Cartesian coordinates of **TS1**$_{trans}$ in the doublet state of **Co(NCTPPSpy)**.

Atom	X	Y	Z
	Angstrom		
Atom	X	Y	Z
N	-1.211140	1.405346	-0.884546
C	-1.200517	2.782257	-0.769089
C	-2.453993	1.078222	-1.375424
C	-2.428262	3.336627	-1.305487
H	-2.652748	4.391570	-1.383045
C	-3.205485	2.280189	-1.678833
H	-4.188746	2.298336	-2.128282
N	1.445709	1.746910	0.108531
C	1.009325	3.027420	0.323082
C	2.735896	1.699412	0.567658
C	3.106438	2.967651	1.157471
H	4.062597	3.188558	1.612013
C	2.028900	3.796245	1.008594
H	1.928725	4.825249	1.325574
N	1.980384	-0.987760	-0.537418
C	3.259587	-0.591590	-0.213086
C	2.067294	-2.330032	-0.828795
C	4.188709	-1.684045	-0.410104
H	5.256803	-1.627598	-0.250545
C	3.447763	-2.762442	-0.798619
C	-0.665543	-1.299863	-1.494548
C	-2.155708	-1.329607	-1.301507
C	-0.319826	-2.696223	-1.086201
C	-1.545691	-3.356144	-0.807797
H	-1.655624	-4.377912	-0.457127
N	-2.613889	-2.563031	-0.933126
C	-0.210752	3.556907	-0.147244
C	-3.002791	-0.230346	-1.441458
C	0.963511	-3.213390	-0.959792
C	3.623747	0.629895	0.371555
Co	0.384476	0.213489	-0.633671
C	-0.952151	7.777022	0.431681
C	-1.719808	6.823246	1.115794
C	-1.482604	5.455768	0.921865
C	-0.467995	5.019047	0.049018
C	0.301895	5.984264	-0.627139
C	0.058728	7.351931	-0.442943
H	-2.502398	7.141747	1.801567
H	-2.075464	4.719979	1.461116
H	1.085074	5.660583	-1.309667
H	0.657549	8.083604	-0.981885

C	-7.256624	-0.834168	-1.858083
C	-6.352044	-1.552742	-2.654578
C	-4.974771	-1.356812	-2.509389
C	-4.470368	-0.427306	-1.577062
C	-5.389355	0.284499	-0.778571
C	-6.767914	0.081555	-0.915154
H	-6.720457	-2.268126	-3.387327
H	-4.279520	-1.918329	-3.127704
H	-5.023515	0.984697	-0.030945
H	-7.458177	0.635066	-0.281418
C	1.495527	-7.490545	-0.727796
C	0.794950	-6.917740	-1.799254
C	0.627033	-5.529052	-1.868648
C	1.166086	-4.685970	-0.875606
C	1.867277	-5.273497	0.197398
C	2.027474	-6.662121	0.272073
H	0.381153	-7.550843	-2.581688
H	0.094017	-5.087617	-2.708025
H	2.265420	-4.638904	0.985748
H	2.561814	-7.097980	1.113979
C	7.611307	1.015320	1.977557
C	7.391556	1.224485	0.608273
C	6.099714	1.101639	0.075516
C	5.016625	0.770333	0.906278
C	5.240736	0.560810	2.279587
C	6.532034	0.682791	2.810690
H	8.222875	1.483649	-0.044671
H	5.930173	1.266823	-0.987001
H	4.397652	0.295713	2.914430
H	6.695440	0.516035	3.873771
S	-0.432089	-1.261567	-3.354037
C	2.295631	2.230293	-4.114427
C	1.835496	1.472924	-5.205498
C	0.989807	0.396368	-4.964855
C	0.623942	0.104314	-3.631191
N	1.056653	0.828115	-2.584526
C	1.879083	1.867490	-2.836669
H	2.958805	3.080221	-4.245975
H	2.133034	1.719563	-6.222691
H	0.609196	-0.218889	-5.775452
H	2.212069	2.423600	-1.968818
C	-0.230222	-0.433798	1.100274
H	-1.165874	-0.992704	1.088730
H	-1.138683	8.839005	0.578845

H	8.613577	1.109937	2.391126
H	1.623134	-8.569783	-0.670674
H	-8.328201	-0.990087	-1.967504
C	0.672870	-0.926076	2.152636
O	0.168740	-2.055821	2.747730
O	1.727212	-0.422743	2.536873
C	-1.188420	1.204104	2.434326
C	-1.632863	0.682546	3.628790
H	-1.874544	1.376864	1.613239
H	-0.258887	1.760409	2.412747
C	0.921536	-2.574659	3.860061
C	0.187908	-3.804211	4.378556
H	1.006246	-1.800214	4.632795
H	1.939489	-2.817964	3.529317
H	0.725850	-4.229524	5.234641
H	-0.826236	-3.542680	4.698710
H	0.113231	-4.571118	3.599557
H	-0.950787	0.707157	4.479554
C	-5.418156	-1.187669	4.433986
C	-4.525916	-0.888482	5.475912
C	-3.294777	-0.287308	5.196321
C	-2.922010	0.042629	3.870932
C	-3.831351	-0.274989	2.831941
C	-5.058707	-0.880241	3.110542
H	-6.374071	-1.661925	4.647432
H	-4.790852	-1.127331	6.504597
H	-2.607664	-0.059721	6.010621
H	-3.562370	-0.072001	1.798720
H	-5.731925	-1.127701	2.291741
H	3.791188	-3.759884	-1.036766

Table S9. Cartesian coordinates of **TS1**$_{cis}$ in the doublet state of **Co(NCTPPSpy)**.

Atom	X	Y	Z
	Angstrom		
N	1.783581	1.290130	-0.528278
C	3.076209	0.812262	-0.644169
C	1.897704	2.652167	-0.379973
C	4.013515	1.917520	-0.683202
H	5.081921	1.823137	-0.820994
C	3.283612	3.056571	-0.509195
H	3.634346	4.079221	-0.484166
N	1.199226	-1.497940	-0.749922
C	2.552750	-1.608199	-0.570968
C	0.712122	-2.778541	-0.798669
C	1.776189	-3.728642	-0.555653
H	1.655112	-4.801814	-0.499451
C	2.926355	-2.999332	-0.421884
H	3.927886	-3.360582	-0.231875
N	-1.543961	-0.828334	-1.183607
C	-1.608947	-2.183195	-1.421522
C	-2.822542	-0.354893	-1.364781
C	-2.932668	-2.548448	-1.880047
H	-3.234305	-3.543282	-2.178217
C	-3.685672	-1.410332	-1.849657
C	-0.923883	1.933487	-0.952144
C	-0.472391	3.115543	-0.139690
C	-2.373965	1.904823	-0.591092
C	-2.609063	3.028177	0.245874
H	-3.555694	3.293209	0.707244
N	-1.504863	3.730263	0.512482
C	3.466507	-0.533489	-0.606695
C	0.848726	3.542921	-0.023060
C	-3.287076	0.924351	-0.958163
C	-0.599789	-3.123850	-1.163588
Co	0.120367	0.197188	-0.788270
C	7.684766	-1.473418	-0.482511
C	7.070436	-0.837782	0.606363
C	5.704631	-0.526794	0.562853
C	4.928915	-0.854462	-0.565169
C	5.552759	-1.500018	-1.649232
C	6.920793	-1.801571	-1.612177
H	7.652177	-0.585580	1.490774
H	5.230656	-0.041899	1.413721
H	4.965207	-1.753602	-2.529330
H	7.388509	-2.291917	-2.463765

C	1.731368	7.440666	1.605029
C	0.778598	7.294610	0.585166
C	0.491451	6.028763	0.063992
C	1.161568	4.883006	0.538542
C	2.113559	5.041967	1.566610
C	2.393609	6.306934	2.097506
H	0.256676	8.167818	0.198464
H	-0.249656	5.921959	-0.723576
H	2.615946	4.166048	1.970838
H	3.122961	6.405486	2.899265
C	-7.506931	1.745949	-0.571792
C	-6.678635	2.605093	-1.308497
C	-5.310614	2.331066	-1.430191
C	-4.746878	1.187101	-0.828932
C	-5.589400	0.331580	-0.089721
C	-6.954975	0.610454	0.040621
H	-7.097057	3.486869	-1.789804
H	-4.673485	2.993193	-2.012634
H	-5.164996	-0.539921	0.403506
H	-7.586962	-0.055165	0.625289
C	-1.664849	-7.306144	-1.240756
C	-0.844768	-6.779074	-2.249068
C	-0.491026	-5.422403	-2.231126
C	-0.962616	-4.574875	-1.213363
C	-1.786731	-5.109039	-0.204383
C	-2.131206	-6.467188	-0.217149
H	-0.480935	-7.421582	-3.048617
H	0.142953	-5.015775	-3.016835
H	-2.133701	-4.453794	0.591717
H	-2.762088	-6.870243	0.572847
S	-0.865401	2.546737	-2.724314
C	1.348512	-0.643920	-4.988283
C	0.845663	0.466045	-5.689032
C	0.162217	1.449993	-4.984288
C	0.002046	1.298556	-3.588055
N	0.488330	0.244097	-2.911398
C	1.142838	-0.704427	-3.612908
H	1.885087	-1.443269	-5.490907
H	0.982374	0.557159	-6.764697
H	-0.250167	2.322680	-5.483092
H	1.512822	-1.542462	-3.034920
C	-0.287632	0.213550	1.121071
H	-0.355037	1.206153	1.564838
H	8.746075	-1.711896	-0.450567

H	-1.936940	-8.359880	-1.252102
H	-8.569368	1.959959	-0.473140
H	1.950050	8.424987	2.014773
C	-1.198923	-0.734609	1.779513
O	-1.994879	-0.101815	2.698642
O	-1.265949	-1.952493	1.608225
C	1.534535	-0.314442	2.443385
C	1.311642	-0.026245	3.772535
H	2.055877	0.409228	1.830377
H	1.532038	-1.337663	2.083002
C	-2.882355	-0.945584	3.459833
C	-3.653615	-0.052211	4.421661
H	-2.292454	-1.697468	3.995684
H	-3.554604	-1.475410	2.772286
H	-4.344642	-0.657391	5.021025
H	-2.967768	0.463622	5.101822
H	-4.233692	0.701853	3.878002
H	1.470297	1.001819	4.100820
C	0.046608	-2.678051	6.909369
C	0.364269	-1.337650	7.180118
C	0.760960	-0.487109	6.143751
C	0.855025	-0.947295	4.807448
C	0.520958	-2.301098	4.550021
C	0.126362	-3.149332	5.587149
H	-0.260185	-3.344224	7.713414
H	0.303599	-0.958530	8.198901
H	1.008066	0.551085	6.362936
H	0.555835	-2.682650	3.533269
H	-0.125572	-4.185219	5.365973
H	-4.723976	-1.286159	-2.125404

Table S10. Cartesian coordinates of $\mathbf{C}_{trans}$ in the doublet state of **Co(NCTPPSpy)**.

Atom	X	Y	Z
	Angstrom		
N	-1.345830	1.404782	-0.795707
C	-1.353549	2.784667	-0.758835
C	-2.610331	1.029970	-1.188552
C	-2.617298	3.288994	-1.262357
H	-2.863068	4.333640	-1.394825
C	-3.398158	2.200982	-1.520066
H	-4.405938	2.177491	-1.911487
N	1.321519	1.820317	0.127166
C	0.873688	3.105581	0.278395
C	2.612173	1.804924	0.590084
C	2.968322	3.102764	1.123843
H	3.920622	3.354437	1.570104
C	1.886284	3.914408	0.928233
H	1.777715	4.957277	1.192825
N	1.905549	-0.904755	-0.497007
C	3.179407	-0.477757	-0.189058
C	2.033063	-2.220653	-0.888477
C	4.140614	-1.521242	-0.474019
H	5.209862	-1.437027	-0.337423
C	3.429920	-2.595373	-0.928393
C	-0.780424	-1.279142	-1.366810
C	-2.242701	-1.361945	-1.046238
C	-0.351637	-2.666212	-1.029513
C	-1.519638	-3.370122	-0.639347
H	-1.561458	-4.399106	-0.294557
N	-2.620311	-2.608268	-0.636872
C	-0.354535	3.601800	-0.208779
C	-3.133279	-0.290426	-1.147451
C	0.959186	-3.137651	-1.029171
C	3.518429	0.747390	0.410579
Co	0.275841	0.258279	-0.549155
C	-1.099597	7.847813	0.131564
C	-1.860817	6.934687	0.875704
C	-1.623020	5.558586	0.759747
C	-0.615094	5.072047	-0.094385
C	0.148552	5.997447	-0.830900
C	-0.094654	7.373344	-0.724332
H	-2.638389	7.291905	1.547994
H	-2.210659	4.855047	1.345728
H	0.926836	5.635119	-1.499439
H	0.499494	8.072936	-1.309097

C	-7.391161	-0.994153	-1.200193
C	-6.533292	-1.752693	-2.011384
C	-5.153771	-1.524610	-1.985943
C	-4.600501	-0.523484	-1.160829
C	-5.473069	0.227650	-0.345517
C	-6.853177	-0.006209	-0.363031
H	-6.940009	-2.524016	-2.662564
H	-4.495945	-2.116351	-2.616330
H	-5.066918	0.980026	0.326365
H	-7.505420	0.578028	0.283168
C	1.623064	-7.397840	-1.261447
C	0.781219	-6.752922	-2.179159
C	0.573742	-5.370859	-2.093450
C	1.210679	-4.602568	-1.095978
C	2.052823	-5.264470	-0.178485
C	2.255007	-6.646817	-0.258847
H	0.288782	-7.324596	-2.963316
H	-0.067877	-4.872534	-2.816590
H	2.531641	-4.694911	0.613189
H	2.900061	-7.139217	0.466261
C	7.508879	1.260939	1.975301
C	7.287732	1.348827	0.593023
C	5.995744	1.180587	0.074465
C	4.911315	0.926146	0.931495
C	5.137244	0.841083	2.318135
C	6.429595	1.006081	2.835006
H	8.118200	1.550081	-0.081083
H	5.825644	1.255757	-0.998086
H	4.294553	0.638348	2.976148
H	6.592979	0.935312	3.908742
S	-0.720078	-1.169633	-3.234607
C	2.143268	2.182592	-4.112104
C	1.602207	1.450422	-5.183144
C	0.720412	0.411836	-4.907458
C	0.395487	0.136530	-3.559728
N	0.901472	0.839574	-2.532793
C	1.763296	1.837464	-2.817982
H	2.840957	2.999561	-4.271255
H	1.869228	1.684244	-6.211791
H	0.283160	-0.187695	-5.701106
H	2.158752	2.374879	-1.964810
C	-0.308501	-0.290676	1.365315
H	-0.990612	-1.132600	1.235413
H	-1.286318	8.916387	0.218353

H	8.511582	1.390681	2.378091
H	1.782908	-8.472417	-1.324695
H	-8.464276	-1.175159	-1.216370
C	0.859831	-0.773470	2.175042
O	0.907861	-2.134367	2.249391
O	1.668535	-0.063471	2.762264
C	-1.032816	0.835187	2.152140
C	-1.425121	0.395070	3.535429
H	-1.915091	1.147671	1.589435
H	-0.367550	1.698372	2.237808
C	1.954649	-2.707719	3.059470
C	1.488617	-4.079722	3.531143
H	2.163608	-2.042001	3.902353
H	2.868676	-2.777321	2.453656
H	2.276909	-4.557070	4.126271
H	0.591926	-3.987616	4.152939
H	1.249245	-4.731659	2.684517
H	-0.699319	0.562595	4.329891
C	-5.035193	-1.583042	4.681350
C	-4.062809	-1.239900	5.642212
C	-2.886108	-0.603516	5.257499
C	-2.629599	-0.272821	3.888461
C	-3.629192	-0.637847	2.932791
C	-4.801841	-1.278765	3.326753
H	-5.952859	-2.084262	4.982262
H	-4.232240	-1.475116	6.691908
H	-2.139809	-0.341483	6.006521
H	-3.465051	-0.434960	1.878764
H	-5.538382	-1.553522	2.573761
H	3.808024	-3.554531	-1.254405

Table S11. Cartesian coordinates of **C*cis*** in the doublet state of **Co(NCTPPSpy)**.

Atom	X (Angstrom)	Y	Z
N	1.626564	1.419672	-0.606363
C	2.930090	1.080350	-0.919925
C	1.631717	2.773226	-0.372592
C	3.745820	2.276836	-1.004024
H	4.787765	2.302134	-1.292130
C	2.946359	3.321496	-0.641307
H	3.201031	4.370984	-0.583063
N	1.280808	-1.402175	-0.776233
C	2.648521	-1.379540	-0.811764
C	0.915042	-2.720505	-0.677049
C	2.090916	-3.555833	-0.558733
H	2.085929	-4.628959	-0.424457
C	3.171775	-2.722090	-0.664384
H	4.220773	-2.982973	-0.627178
N	-1.533795	-0.985386	-0.948990
C	-1.500527	-2.354975	-1.083437
C	-2.840263	-0.612179	-1.183032
C	-2.795539	-2.848953	-1.497851
H	-3.026593	-3.884069	-1.710644
C	-3.620359	-1.763176	-1.585505
C	-1.112442	1.838567	-0.813050
C	-0.743784	3.011344	0.047730
C	-2.557245	1.700402	-0.479196
C	-2.873092	2.765966	0.405727
H	-3.839727	2.946887	0.866798
N	-1.815854	3.515091	0.731492
C	3.441848	-0.223243	-0.992266
C	0.544254	3.537455	0.139088
C	-3.395385	0.655687	-0.863240
C	-0.398670	-3.193251	-0.835588
Co	0.064030	0.179165	-0.712262
C	7.681511	-0.800831	-1.611162
C	7.218270	-0.195302	-0.433909
C	5.845088	0.000100	-0.234316
C	4.911679	-0.413753	-1.203569
C	5.386370	-1.030199	-2.376909
C	6.759757	-1.216380	-2.583357
H	7.924133	0.122557	0.330879
H	5.490555	0.460201	0.685523
H	4.675157	-1.351741	-3.135042
H	7.108781	-1.685045	-3.501445

C	1.264687	7.345827	2.035525
C	0.220586	7.201382	1.109141
C	-0.015916	5.965837	0.498017
C	0.795197	4.849329	0.787836
C	1.839197	5.006482	1.723721
C	2.070306	6.240177	2.343842
H	-0.411800	8.052727	0.864641
H	-0.827839	5.862077	-0.215960
H	2.455751	4.149393	1.985430
H	2.874445	6.335614	3.070961
C	-7.660373	1.325452	-0.746281
C	-6.811697	2.216023	-1.419441
C	-5.430957	1.985359	-1.450348
C	-4.869930	0.854935	-0.819329
C	-5.735007	-0.031511	-0.144082
C	-7.114103	0.202944	-0.105796
H	-7.224004	3.088549	-1.922360
H	-4.777607	2.671350	-1.984931
H	-5.322163	-0.895333	0.369911
H	-7.762238	-0.487711	0.430224
C	-1.156084	-7.423848	-0.447767
C	-0.474387	-6.942055	-1.574950
C	-0.218093	-5.570237	-1.709060
C	-0.650601	-4.663694	-0.724939
C	-1.336462	-5.152482	0.403667
C	-1.582556	-6.525035	0.541751
H	-0.143096	-7.631753	-2.348912
H	0.306789	-5.198976	-2.587269
H	-1.648996	-4.449261	1.172591
H	-2.105143	-6.892429	1.422900
S	-1.055351	2.542361	-2.551800
C	0.822477	-0.713226	-5.016981
C	0.286474	0.409854	-5.670569
C	-0.300069	1.412997	-4.907344
C	-0.323412	1.270379	-3.501576
N	0.204447	0.208301	-2.870438
C	0.753804	-0.763515	-3.627235
H	1.279603	-1.531136	-5.566181
H	0.317165	0.493144	-6.755087
H	-0.743215	2.291476	-5.368111
H	1.153546	-1.611630	-3.084509
C	-0.021538	0.125887	1.358196
H	-0.298047	1.145329	1.631471
H	8.748102	-0.949121	-1.768160

H	-1.351881	-8.489149	-0.341253
H	-8.733365	1.504729	-0.718473
H	1.444516	8.306492	2.514453
C	-1.089132	-0.799927	1.859470
O	-2.240223	-0.140405	2.169764
O	-0.972548	-2.010893	2.016835
C	1.323554	-0.241031	2.036038
C	1.324086	0.157368	3.484899
H	2.131346	0.298335	1.532423
H	1.516922	-1.309227	1.918781
C	-3.311556	-0.925896	2.734753
C	-3.149246	-1.093633	4.244189
H	-3.348410	-1.900412	2.236616
H	-4.220616	-0.360871	2.503502
H	-4.006984	-1.644479	4.650853
H	-2.237031	-1.650477	4.476176
H	-3.099848	-0.117569	4.738803
H	1.339641	1.230452	3.683348
C	1.280860	-2.330257	6.958618
C	1.360722	-0.927891	7.071886
C	1.360621	-0.128746	5.932520
C	1.286419	-0.697218	4.619432
C	1.191278	-2.123057	4.530832
C	1.191808	-2.912258	5.679138
H	1.282033	-2.955299	7.849300
H	1.423211	-0.466319	8.056192
H	1.423069	0.954467	6.029874
H	1.087309	-2.590609	3.556398
H	1.115893	-3.994453	5.584458
H	-4.657094	-1.736881	-1.891439

Table S12. Cartesian coordinates of **$TS2_{trans}$** in the doublet state of **Co(NCTPPSpy)**.

	Angstrom		
Atom	X	Y	Z
N	-1.333573	1.467334	-0.726144
C	-1.303979	2.846218	-0.675367
C	-2.610838	1.132213	-1.124301
C	-2.554989	3.391902	-1.169088
H	-2.771413	4.444294	-1.291961
C	-3.365879	2.329424	-1.440776
H	-4.372446	2.340913	-1.836054
N	1.363218	1.798710	0.141384
C	0.951281	3.092603	0.323954
C	2.659672	1.741249	0.585629
C	3.057210	3.019597	1.139434
H	4.021376	3.238446	1.577508
C	1.994274	3.862326	0.975400
H	1.917041	4.901816	1.263467
N	1.865281	-0.934177	-0.530138
C	3.153291	-0.548055	-0.230743
C	1.953255	-2.246431	-0.950778
C	4.084273	-1.611687	-0.548049
H	5.157260	-1.558263	-0.425552
C	3.340719	-2.656322	-1.016902
C	-0.850562	-1.233847	-1.379798
C	-2.297712	-1.271693	-1.013694
C	-0.447271	-2.625583	-1.053521
C	-1.616191	-3.294606	-0.608451
H	-1.671674	-4.317402	-0.247020
N	-2.694863	-2.501191	-0.570194
C	-0.272473	3.628912	-0.131839
C	-3.165254	-0.174201	-1.094357
C	0.854412	-3.131374	-1.089371
C	3.533000	0.660407	0.381033
Co	0.276885	0.279406	-0.568068
C	-0.888935	7.892386	0.260876
C	-1.682762	6.993710	0.988302
C	-1.486437	5.612568	0.855931
C	-0.488623	5.104517	0.002082
C	0.307077	6.016235	-0.718056
C	0.106081	7.397463	-0.595054
H	-2.453334	7.366266	1.660426
H	-2.099861	4.920777	1.429345
H	1.077199	5.638490	-1.387372
H	0.725106	8.085528	-1.167618

C	-7.440066	-0.785097	-1.115813
C	-6.600272	-1.584895	-1.906005
C	-5.216250	-1.385717	-1.890873
C	-4.636780	-0.374301	-1.095869
C	-5.492872	0.417741	-0.300973
C	-6.877902	0.214973	-0.309381
H	-7.025024	-2.365251	-2.534535
H	-4.574117	-2.008427	-2.507088
H	-5.070186	1.183276	0.345394
H	-7.515920	0.834468	0.318151
C	1.378749	-7.405506	-1.421735
C	0.525345	-6.716966	-2.295968
C	0.364727	-5.331161	-2.177584
C	1.060242	-4.601139	-1.190046
C	1.912348	-5.307611	-0.315556
C	2.068617	-6.693588	-0.428775
H	-0.012452	-7.257639	-3.072334
H	-0.285799	-4.799712	-2.868329
H	2.435156	-4.769624	0.470078
H	2.723040	-7.219533	0.263734
C	7.559405	1.044348	1.893754
C	7.321706	1.144648	0.515018
C	6.018085	1.018723	0.013941
C	4.937620	0.794416	0.884550
C	5.180683	0.697751	2.267666
C	6.484495	0.820248	2.767398
H	8.148561	1.323016	-0.169941
H	5.835612	1.104060	-1.055788
H	4.340727	0.521056	2.936860
H	6.660214	0.741049	3.838650
S	-0.809111	-1.089421	-3.241153
C	2.103277	2.209799	-4.200917
C	1.514291	1.489011	-5.254873
C	0.618547	0.467500	-4.955799
C	0.330118	0.198016	-3.598672
N	0.883034	0.887633	-2.591592
C	1.754340	1.868564	-2.895878
H	2.811833	3.012788	-4.382602
H	1.755093	1.719246	-6.290888
H	0.145661	-0.119783	-5.738346
H	2.183181	2.394447	-2.049265
C	-0.333432	-0.345303	1.675799
H	-1.011348	-1.159583	1.431153
H	-1.043018	8.965078	0.360292

H	8.571181	1.141323	2.282911
H	1.502718	-8.483023	-1.510588
H	-8.516990	-0.942309	-1.125482
C	0.907529	-0.807510	2.346017
O	1.047829	-2.160834	2.274601
O	1.713053	-0.085021	2.926881
C	-0.990488	0.869282	2.318181
C	-1.384384	0.214598	3.592528
H	-1.829072	1.233888	1.726639
H	-0.268740	1.672696	2.472542
C	2.209953	-2.740264	2.903449
C	1.821855	-4.106629	3.456250
H	2.564051	-2.071343	3.693002
H	3.002290	-2.822830	2.147772
H	2.700669	-4.593471	3.896814
H	1.055639	-4.006395	4.232500
H	1.423664	-4.754623	2.668399
H	-0.615629	0.164839	4.361193
C	-5.095860	-1.670018	4.554696
C	-4.049757	-1.615296	5.494030
C	-2.836877	-1.015274	5.159472
C	-2.632041	-0.430806	3.876039
C	-3.702498	-0.501591	2.939840
C	-4.908458	-1.114400	3.276173
H	-6.039472	-2.146792	4.811968
H	-4.185444	-2.047620	6.483836
H	-2.028273	-0.980615	5.888307
H	-3.572902	-0.097970	1.940240
H	-5.703724	-1.170480	2.535683
H	3.690887	-3.616472	-1.370346

Table S13. Cartesian coordinates of **TS2*cis*** in the doublet state of **Co(NCTPPSpy)**.

	Angstrom		
Atom	X	Y	Z
N	1.936410	1.131738	-0.427382
C	3.187195	0.583086	-0.639277
C	2.145994	2.470411	-0.184353
C	4.193314	1.629065	-0.647907
H	5.244539	1.483116	-0.855439
C	3.550094	2.793850	-0.344915
H	3.969060	3.787692	-0.263260
N	1.137955	-1.583933	-0.703233
C	2.488271	-1.795066	-0.619574
C	0.548669	-2.822628	-0.673712
C	1.551880	-3.849047	-0.480807
H	1.353278	-4.907837	-0.382555
C	2.762371	-3.210619	-0.465621
H	3.744457	-3.648067	-0.346228
N	-1.553170	-0.700905	-1.081303
C	-1.736754	-2.054758	-1.246723
C	-2.756748	-0.112175	-1.417665
C	-3.057531	-2.323805	-1.775952
H	-3.436734	-3.304527	-2.030472
C	-3.679212	-1.115376	-1.911099
C	-0.685293	2.026610	-0.867717
C	-0.189710	3.083690	0.065397
C	-2.147896	2.109286	-0.629092
C	-2.348672	3.162075	0.302004
H	-3.300497	3.467084	0.727497
N	-1.206892	3.714248	0.728013
C	3.478535	-0.789047	-0.697428
C	1.160517	3.395637	0.257422
C	-3.117108	1.225974	-1.115352
C	-0.807687	-3.066711	-0.944986
Co	0.201345	0.179139	-0.730655
C	7.601336	-2.068670	-0.960185
C	7.151085	-1.372404	0.171015
C	5.817666	-0.949888	0.254836
C	4.908441	-1.222535	-0.785310
C	5.369453	-1.929973	-1.912351
C	6.704918	-2.344045	-2.003283
H	7.835906	-1.160038	0.989713
H	5.472454	-0.418861	1.139327
H	4.679128	-2.142486	-2.726032
H	7.044350	-2.880657	-2.887167

C	2.318880	7.035063	2.268046
C	1.315703	7.054001	1.286827
C	0.938226	5.873428	0.639222
C	1.564328	4.647109	0.946161
C	2.568609	4.641527	1.937844
C	2.940524	5.821019	2.593866
H	0.825662	7.990578	1.027635
H	0.159814	5.896793	-0.117844
H	3.042397	3.702081	2.213229
H	3.710086	5.790460	3.362902
C	-7.186155	2.655948	-1.369000
C	-6.124645	3.405409	-1.896194
C	-4.812067	2.926694	-1.807245
C	-4.530238	1.684327	-1.198859
C	-5.608638	0.942537	-0.671175
C	-6.920003	1.423654	-0.753220
H	-6.318238	4.360924	-2.379749
H	-3.994123	3.504422	-2.231417
H	-5.415777	-0.005046	-0.176163
H	-7.734472	0.839308	-0.329346
C	-2.257056	-7.129842	-0.767582
C	-1.485949	-6.714649	-1.863191
C	-1.006522	-5.398457	-1.927745
C	-1.300465	-4.480300	-0.904502
C	-2.074870	-4.902358	0.192911
C	-2.546476	-6.220442	0.261012
H	-1.257912	-7.412873	-2.666339
H	-0.411101	-5.077567	-2.780534
H	-2.281201	-4.194983	0.993408
H	-3.137962	-6.537430	1.117974
S	-0.379071	2.714972	-2.578964
C	1.190314	-0.719487	-5.034338
C	0.881195	0.492768	-5.676153
C	0.387749	1.550536	-4.918812
C	0.226838	1.366905	-3.526880
N	0.532134	0.217365	-2.909424
C	0.995819	-0.802055	-3.656921
H	1.568241	-1.577181	-5.583363
H	1.015430	0.604995	-6.750250
H	0.122725	2.500308	-5.375487
H	1.217655	-1.716628	-3.116463
C	-0.092595	0.067642	1.649453
H	-0.252369	1.137736	1.752223
H	8.637620	-2.394111	-1.027295

H	-2.627112	-8.152084	-0.715422
H	-8.206877	3.027533	-1.434727
H	2.608315	7.953617	2.775106
C	-1.299638	-0.742646	1.918680
O	-2.418756	0.024443	2.018536
O	-1.320483	-1.964601	2.046082
C	1.195537	-0.466244	2.258838
C	0.918770	-0.062770	3.663356
H	2.069664	0.035228	1.842149
H	1.287779	-1.543575	2.117992
C	-3.654988	-0.665389	2.295679
C	-3.828939	-0.935210	3.789053
H	-3.686453	-1.597728	1.722154
H	-4.433392	0.013000	1.932777
H	-4.807140	-1.398354	3.970854
H	-3.050464	-1.611181	4.153435
H	-3.778337	-0.000770	4.358469
H	1.023529	1.000800	3.876536
C	-0.320868	-2.430546	6.993880
C	-0.184645	-1.034172	7.109618
C	0.196103	-0.274797	6.006001
C	0.464114	-0.884100	4.743802
C	0.309976	-2.297825	4.645027
C	-0.073657	-3.049652	5.755864
H	-0.617535	-3.025488	7.855417
H	-0.378181	-0.546155	8.063157
H	0.298496	0.805489	6.100053
H	0.477711	-2.791724	3.693704
H	-0.183944	-4.128348	5.660453
H	-4.665223	-0.915458	-2.307274

Table S14. Cartesian coordinates of **PC*trans*** in the doublet state of **Co(NCTPPSpy)**.

Atom	X	Y	Z
	Angstrom		
C	-0.530810	3.399474	-3.081245
C	0.177716	2.678248	-4.062571
C	0.219966	3.194469	-5.371324
C	-0.426771	4.395253	-5.694149
C	-1.129233	5.104018	-4.709365
C	-1.177693	4.598877	-3.400928
C	0.881112	1.393305	-3.766492
C	0.478537	0.484244	-2.639572
C	1.767380	1.271569	-2.506234
C	3.048503	0.531754	-2.653064
O	3.190288	-0.509862	-3.278826
Co	-0.419161	-0.542025	0.727761
N	-2.371548	-0.578315	0.271790
C	-3.070651	-1.710028	-0.102543
C	-4.498854	-1.485703	0.022704
C	-4.659430	-0.186550	0.402179
C	-3.331547	0.384151	0.519585
C	-2.513954	-2.887759	-0.622256
C	-3.434761	-3.956203	-1.125740
C	-3.486840	-5.214576	-0.496369
C	-4.347710	-6.215981	-0.965234
C	-5.163991	-5.979098	-2.081044
C	-5.115073	-4.731359	-2.719756
C	-4.260966	-3.727929	-2.242767
C	-3.086252	1.766028	0.711397
C	-4.175717	2.759935	0.528061
C	-5.028404	2.721337	-0.594443
C	-6.044474	3.670938	-0.757494
C	-6.232263	4.675600	0.202790
C	-5.386164	4.728247	1.321052
C	-4.364460	3.785751	1.476840
N	-0.709715	-1.500860	2.640660
C	-0.622386	-2.820013	2.895852
C	-0.824791	-3.370298	4.159968
C	-1.133755	-2.498120	5.219555
C	-1.222143	-1.131689	4.969521
C	-1.000291	-0.672108	3.651902
S	-1.123574	1.060184	3.353525
C	-0.697736	1.333072	1.553262
C	-1.786208	2.195140	1.001281
N	-1.372614	3.475624	0.750347

C	-0.076749	3.533755	1.074328
C	0.440222	2.291309	1.528503
C	1.781617	1.996352	1.768381
C	2.253480	0.669250	1.623042
C	3.617695	0.269497	1.895895
C	3.767696	-0.988530	1.388842
C	2.487843	-1.363083	0.823063
N	1.558538	-0.362505	1.021105
C	2.287458	-2.542266	0.085932
C	1.043584	-2.934057	-0.434478
C	0.824686	-4.148108	-1.192793
C	-0.525006	-4.253349	-1.378831
C	-1.124071	-3.102667	-0.732736
N	-0.151998	-2.293874	-0.199318
O	4.065997	1.160413	-2.017338
C	5.363781	0.522590	-2.121962
C	6.384463	1.428419	-1.450111
C	2.733852	3.108103	2.047482
C	2.484974	3.996353	3.113077
C	3.358154	5.059358	3.377437
C	4.486228	5.262522	2.568947
C	4.733006	4.394380	1.494341
C	3.867419	3.324265	1.237379
C	3.471581	-3.422500	-0.187084
C	4.105209	-3.382632	-1.443283
C	5.207689	-4.208951	-1.705130
C	5.690374	-5.082394	-0.719144
C	5.063208	-5.124153	0.534846
C	3.961155	-4.298119	0.798056
H	0.495020	4.442247	0.907700
H	4.354900	0.880717	2.398789
H	4.653752	-1.608899	1.388373
H	1.600575	-4.822611	-1.528660
H	-1.067476	-5.027258	-1.904563
H	-5.264225	-2.228877	-0.154361
H	-5.580873	0.344449	0.598392
H	1.613009	3.837328	3.743878
H	3.157564	5.728092	4.212240
H	5.161832	6.091822	2.769612
H	5.594964	4.556497	0.849913
H	4.045766	2.671490	0.385972
H	3.739049	-2.700363	-2.208611
H	5.688096	-4.168399	-2.680971
H	6.545198	-5.723766	-0.925254

H	5.428555	-5.799440	1.306317
H	3.472568	-4.335662	1.770017
H	-2.859507	-5.402282	0.372577
H	-4.381174	-7.178916	-0.459182
H	-5.829602	-6.757647	-2.448648
H	-5.739355	-4.538703	-3.590090
H	-4.221264	-2.764376	-2.746532
H	-4.873701	1.962551	-1.358134
H	-6.682818	3.630745	-1.638050
H	-7.023783	5.412296	0.078384
H	-5.521521	5.505751	2.070520
H	-3.711474	3.832457	2.344436
H	-1.454734	-0.424256	5.761013
H	-1.300183	-2.880912	6.224570
H	-0.743200	-4.443284	4.307999
H	-0.380314	-3.447141	2.042142
H	1.786782	2.128876	-1.841174
H	5.595624	0.365747	-3.181317
H	5.313487	-0.458424	-1.636959
H	7.381857	0.980534	-1.530226
H	6.409188	2.413636	-1.928429
H	6.153517	1.559783	-0.387695
H	0.587339	-0.584100	-2.797625
H	1.296976	0.888984	-4.635913
H	-0.350694	0.784249	-2.005624
H	0.762302	2.650043	-6.143341
H	-0.383037	4.775550	-6.713291
H	-1.634014	6.035691	-4.957428
H	-1.723261	5.132671	-2.625427
H	-0.592741	3.033926	-2.058227

Table S15. Cartesian coordinates of **PC**$_{cis}$ in the doublet state of **Co(NCTPPSpy)**.

Atom	X	Y	Z
	Angstrom		
C	-2.603932	-3.000088	3.673768
C	-2.254909	-1.819279	4.356575
C	-2.602924	-1.706639	5.717612
C	-3.281763	-2.736442	6.380151
C	-3.625368	-3.907761	5.689553
C	-3.282024	-4.032005	4.335561
C	-1.510445	-0.683976	3.726845
C	-0.852177	-0.715279	2.376108
C	-2.054272	0.183089	2.552692
C	-3.336715	-0.182618	1.890304
O	-3.574524	-1.234080	1.314797
Co	0.793113	0.197198	-0.761728
N	2.560329	0.047345	0.174107
C	3.377308	-1.067325	0.134674
C	4.720018	-0.722553	0.562625
C	4.685916	0.586748	0.941837
C	3.329719	1.051821	0.727415
C	2.964935	-2.375125	-0.159793
C	3.946808	-3.494622	-0.004837
C	4.348242	-4.252804	-1.121500
C	5.265341	-5.303206	-0.983539
C	5.787623	-5.622591	0.278450
C	5.388626	-4.879144	1.398885
C	4.479642	-3.822191	1.256713
C	2.853156	2.315921	1.158536
C	3.630655	3.119232	2.137059
C	4.182801	2.531705	3.294451
C	4.903620	3.299508	4.217138
C	5.093236	4.671564	3.997657
C	4.544980	5.268980	2.852307
C	3.815519	4.502874	1.937340
N	1.680878	0.334496	-2.725031
C	1.868610	-0.680558	-3.589262
C	2.466396	-0.522955	-4.838147
C	2.892682	0.764988	-5.209827
C	2.697867	1.824448	-4.328191
C	2.080874	1.561235	-3.084616
S	1.845407	2.911561	-1.977535
C	0.900854	2.242284	-0.506772
C	1.611015	2.767468	0.699678
N	0.900369	3.739794	1.349629

C	-0.242242	3.900329	0.674495
C	-0.364943	3.018876	-0.432450
C	-1.511199	2.817481	-1.204053
C	-1.758737	1.548385	-1.782769
C	-2.910326	1.249253	-2.608504
C	-2.962552	-0.109045	-2.732864
C	-1.826199	-0.636924	-2.006760
N	-1.069415	0.388151	-1.479714
C	-1.588358	-2.013481	-1.849368
C	-0.417928	-2.538073	-1.275576
C	-0.148677	-3.951020	-1.109342
C	1.140375	-4.052508	-0.664607
C	1.646232	-2.700280	-0.544523
N	0.672217	-1.794752	-0.883454
O	-4.242678	0.816162	2.012701
C	-5.544005	0.569639	1.429851
C	-6.426620	1.764412	1.759895
C	-2.516001	3.911489	-1.307039
C	-2.100390	5.199704	-1.703310
C	-3.017883	6.253607	-1.796062
C	-4.368141	6.042936	-1.480659
C	-4.791042	4.769482	-1.070414
C	-3.874974	3.714511	-0.986228
C	-2.651811	-2.969291	-2.297221
C	-3.860522	-3.055737	-1.579150
C	-4.859641	-3.951830	-1.983858
C	-4.670387	-4.764101	-3.111922
C	-3.469066	-4.681102	-3.831425
C	-2.464447	-3.793220	-3.421969
H	-1.003371	4.588554	1.031380
H	-3.581349	1.984051	-3.032173
H	-3.686759	-0.704792	-3.271980
H	-0.853317	-4.748331	-1.302998
H	1.692484	-4.950097	-0.420919
H	5.565464	-1.396924	0.570187
H	5.497033	1.195909	1.316891
H	-1.055814	5.362419	-1.959532
H	-2.679532	7.237126	-2.116193
H	-5.081549	6.861924	-1.548814
H	-5.834160	4.600644	-0.809332
H	-4.204276	2.736089	-0.646025
H	-3.998362	-2.435829	-0.695988
H	-5.785136	-4.017136	-1.414686
H	-5.449609	-5.455929	-3.426414

H	-3.313684	-5.305248	-4.709399
H	-1.534709	-3.727847	-3.984048
H	3.949230	-4.006742	-2.103406
H	5.571009	-5.871009	-1.860285
H	6.495432	-6.441998	0.387501
H	5.781577	-5.122887	2.384026
H	4.165645	-3.255693	2.130823
H	4.021670	1.473044	3.484957
H	5.310673	2.827746	5.109514
H	5.655927	5.269021	4.712561
H	4.684210	6.333550	2.673861
H	3.392096	4.972344	1.053600
H	3.006969	2.835522	-4.579648
H	3.365303	0.937770	-6.174754
H	2.591960	-1.378469	-5.495552
H	1.520976	-1.652556	-3.250438
H	-1.901271	1.252089	2.666288
H	-5.947949	-0.361489	1.842555
H	-5.427757	0.433184	0.347629
H	-7.421266	1.624009	1.320640
H	-6.539485	1.877194	2.843240
H	-5.997509	2.690668	1.362668
H	-1.009387	-1.589502	1.752741
H	-1.009147	-0.056460	4.461239
H	0.118106	-0.238724	2.269081
H	-2.335278	-0.802210	6.263144
H	-3.537594	-2.627352	7.432586
H	-4.149263	-4.713454	6.200469
H	-3.541059	-4.937940	3.790590
H	-2.355361	-3.114611	2.623175

Table S16. Cartesian coordinates of **TSi** in the doublet state of **Co(NCTPPSpy)**.

Atom	X	Y	Z
	Angstrom		
C	-5.638537	-0.241891	3.361968
C	-5.880839	-0.890658	4.587647
C	-4.787092	-1.348007	5.350140
C	-3.484517	-1.163439	4.897038
C	-3.209691	-0.510813	3.651548
C	-4.337874	-0.052991	2.898056
C	-1.865619	-0.336893	3.227063
C	-1.414542	0.310073	1.946850
C	-0.128654	-0.339214	1.393781
C	1.018707	0.003111	2.292133
O	1.219947	1.090571	2.822699
Co	0.269077	0.128723	-0.584821
N	0.635467	0.661305	-2.644098
C	0.970020	-0.333478	-3.483401
C	1.234099	-0.119015	-4.854981
C	1.152811	1.175112	-5.356652
C	0.824959	2.222649	-4.478593
C	0.580415	1.912998	-3.143324
S	1.062503	-1.964564	-2.863914
C	0.945412	-1.743941	-1.002534
C	0.098657	-2.908394	-0.578008
N	0.827001	-3.882142	0.048175
C	2.092689	-3.456888	0.089302
C	2.270853	-2.160328	-0.465249
C	-1.273281	-2.997232	-0.807607
C	-2.012574	-4.266418	-0.592026
C	-1.514781	-5.487893	-1.089973
C	-2.215288	-6.681461	-0.887765
C	-3.420828	-6.682011	-0.169232
C	-3.918529	-5.476076	0.345105
C	-3.223087	-4.279782	0.132525
C	3.420670	-1.375155	-0.402570
C	4.743091	-2.040470	-0.242863
C	5.094097	-3.087967	-1.120697
C	6.323185	-3.747796	-0.999733
C	7.223985	-3.380592	0.010378
C	6.882583	-2.348233	0.897075
C	5.657046	-1.684423	0.771174
C	-1.971679	-1.830049	-1.231082
N	-1.546256	-0.533409	-1.079271
C	-2.599889	0.266584	-1.482396

C	-3.666442	-0.557383	-2.019902
C	-3.288749	-1.855509	-1.835346
C	-2.709323	1.647061	-1.257743
C	-3.973370	2.349514	-1.644100
C	-5.202954	2.032970	-1.035399
C	-6.376248	2.708880	-1.395320
C	-6.341875	3.709178	-2.377677
C	-5.122778	4.032038	-2.991603
C	-3.948874	3.362321	-2.622242
C	-1.703869	2.409999	-0.617774
N	-0.431024	1.981549	-0.358091
C	0.233830	3.044702	0.198157
C	-0.671149	4.159617	0.375676
C	-1.873102	3.771690	-0.153305
C	1.622984	3.091595	0.401500
C	2.218568	4.325700	1.002680
C	2.333227	5.526127	0.279898
C	2.898568	6.663141	0.874497
C	3.344329	6.614240	2.203496
C	3.224547	5.421141	2.932115
C	2.669644	4.280865	2.335765
C	3.308108	0.041265	-0.379688
N	2.129647	0.739446	-0.223037
C	2.490892	2.034108	0.074653
C	3.929218	2.176306	0.024368
C	4.434870	0.945804	-0.288211
O	1.836970	-1.066575	2.513622
C	2.961099	-0.856973	3.392239
C	2.569185	-1.011701	4.860630
H	-4.579506	-0.185887	-2.464136
H	-3.827400	-2.754262	-2.103349
H	-0.416478	5.106275	0.832511
H	-2.791550	4.340474	-0.206329
H	4.467698	3.100706	0.184766
H	2.856161	-4.048830	0.585954
H	-7.315004	2.455694	-0.906638
H	-5.236868	1.263692	-0.267064
H	-3.006473	3.614211	-3.104148
H	-5.084965	4.804468	-3.757252
H	-1.819680	-7.612872	-1.288384
H	-0.581017	-5.494677	-1.645007
H	-3.604009	-3.353976	0.558177
H	-4.842803	-5.466003	0.919400
H	6.577807	-4.545358	-1.694913

H	4.403563	-3.365922	-1.913801
H	5.397605	-0.898543	1.475185
H	7.568845	-2.064188	1.692539
H	2.990150	7.583716	0.301231
H	1.991567	5.565696	-0.752822
H	2.558376	3.355478	2.896892
H	3.560721	5.378244	3.966359
H	0.762322	3.253371	-4.815091
H	1.352469	1.369027	-6.408592
H	1.502639	-0.958575	-5.490171
H	0.325641	2.690891	-2.433779
H	-0.242627	-1.423971	1.383976
H	-7.253613	4.231921	-2.659892
H	3.779188	7.497786	2.666981
H	8.178553	-3.893988	0.107679
H	-3.961964	-7.612349	-0.006877
H	-2.212714	0.251400	1.205359
H	-1.222739	1.377561	2.126818
H	3.386517	0.135079	3.207314
H	3.685043	-1.625946	3.103669
H	3.459103	-0.917868	5.495756
H	1.854848	-0.235965	5.151103
H	2.120880	-1.995197	5.039856
H	-1.089169	-0.685265	3.908347
H	-6.898970	-1.033934	4.943254
H	-4.962167	-1.849736	6.300519
H	-2.646769	-1.522105	5.493540
H	-4.176019	0.462385	1.954808
H	-6.478620	0.120654	2.771098
H	5.469233	0.670687	-0.441123
